# Relationship between left ventricular mechanical synchrony and left ventricular systolic function: a CZT-SPECT analysis

**DOI:** 10.1186/s12872-022-02863-8

**Published:** 2022-09-22

**Authors:** Qiting Sun, Ruiliang Huang, Songhai Fu, Chen Wu, Xuliang Guo, Tianliang Li, Yuehong Hou, Fei Wang, Rui Xi, Sijin Li

**Affiliations:** 1 Department of Nuclear Medicine, First Hospital of Shanxi Medical University, Collaborative Innovation Center for Molecular Imaging of Precision Medicine, 85 Jiefang Nan Road, Taiyuan, 030001 Shanxi Province China; 2 Department of Radiology, Shanxi Cardiovascular Hospital, Taiyuan, China; 3 Department of Nuclear Medicine, Shanxi Cardiovascular Hospital, Taiyuan, China; 4 Department of Ultrasound, Shanxi Cardiovascular Hospital, Taiyuan, China; 5 Department of Cardiology, Shanxi Cardiovascular Hospital, Taiyuan, China

**Keywords:** Systolic function, Mechanical synchronization, CZT-SPECT

## Abstract

**Background:**

CZT-SPECT has good agreement in the evaluation of mechanical synchronization compared with conventional SPECT. The aim of this study was to evaluate the correlation between left ventricular mechanical contraction synchrony and left ventricular systolic function by gated myocardial perfusion imaging (GMPI) using cadmium–zine–telluride (CZT) single photon emission computed tomography (SPECT).

**Methods:**

This retrospective study involved 371 patients (239 males and 132 females, mean age 61.06 ± 11.78 years old) who underwent GMPI at the Nuclear Medicine Department of Shanxi Cardiovascular Hospital from January 2020 to August 2020. Systolic synchrony parameters and left ventricular systolic function parameters were calculated via Emory Cardiac Toolbox, including PP, PSD, PHB, HS, HK, EDV, ESV, and LVEF. Based on LVEF value, patients were divided into the severe reduction group (group 1, 127 cases, EF < 35%), moderate reduction group (group 2, 47 cases, 35% ≤ EF < 45%), mild reduction group (group 3, 50 cases, 45% ≤ EF < 50%) and normal group (group 4, 147 cases, EF ≥ 50%). Differences in PP, PSD, PHB, HS and HK among the four groups were compared using one-way ANOVA. Differences between two groups were compared using LSD-t test. The correlation among functional and mechanical contraction synchrony factors were analyzed using Pearson test.

**Results:**

PP, PSD, PHB, HS and HK were significantly different among the four groups (F = 5.20, 188.72, 202.88, 171.05, 101.36, *P* < 0.001). Pairwise comparison tests showed significant differences in PSD and PHB in each two groups, and HS and HK in each two groups except for group 2 and 3 (t = 0.28 and 0.39, both *P* > 0.001). PP was significantly higher in group 1, relative to group 3 (t = 2.43, *P* < 0.001) and group 4 (t = 3.67, *P* < 0.001). Pearson correlation analysis revealed that LVEF negatively correlates with PP, PSD, PHB (r = 0.194, − 0.790, − 0.799, all *P* < 0.001). HS and HK showed positive correlation for LVEF (r = 0.778 and 0.795, *P* < 0.001), PSD, PHB and ESV were had good positive correlation (r = 0.778, 0.795, *P* < 0.001), PSD, PHB and EDV had good positive correlation (r = 0.722, 0.732, *P* < 0.001). However, PP had poor correlation with EDV (r = 0.095, *P* > 0.001). HS and HK were negatively correlated with EDV and ESV (r =  − 0.700 to − 0.594, *P* < 0.001).

**Conclusion:**

CZT SPECT GMPI provided left ventricular mechanical contraction synchrony parameters that correlated well with left ventricular systolic function. Worse left ventricular mechanical contraction synchrony lead to decreased LVEF, making the systolic synchrony parameters valuable in the prediction of left ventricular systolic function.

## Introduction

Techniques that detect cardiac mechanical contraction synchrony [1–3] include M-mode ultrasound, tissue Doppler imaging (TDI), speckle tracking imaging (STI) technology, real time three dimensional echocardiography (RT-3DE), gated myocardial perfusion imaging (GMPI), and cardiac magnetic resonance imaging (CMR) [4]. GMPI phase analysis quantitatively evaluates left ventricular contraction synchrony and the important information such as myocardial blood perfusion, ventricular wall motion, left ventricular systolic function, and left ventricular mechanical contraction synchrony can be obtained through a “one-stop” collection. Due to the outstanding application values on cardiac mechanical contraction synchrony, GMPI phase analysis is widely used in CRT electrode implantation guidance and prognosis prediction [5, 6]. The novel cardiology focused SEPCT utilizes solid-state cadmium zinc telluride (CZT) crystal as a detector, and has numerous advantages including high sensitivity, high spatial resolution, short acquisition time, and less radiation dose [7]. However, research on left ventricular mechanical contraction synchrony is limited. Left ventricular ejection fraction (LVEF) is a powerful predictor of cardiac mortality for the patients with heart failure, mechanical dyssynchrony is also important for the patients with heart failure. Here, we analyzed correlation between left ventricular function parameters and quantitative parameters of mechanical contraction synchrony using CZT SPECT phase analysis software to explore the role of mechanical systolic synchrony parameters for predicting the prognosis of patients with heart failure in the future, and evaluated the application of CZT SPECT in mechanical contraction synchrony.

## Methods

### Patient characteristics

We retrospectively enrolled 371 patients (239 males and 132 females, mean age 61.06 ± 11.78 years old) who underwent CZT SPECT resting GMPI at the Nuclear Medicine Department of Shanxi Cardiovascular Hospital from January 2020 to August 2020. Based on LVEF value, patients were divided into the severe reduction group (group 1, 127 cases, EF < 35%), moderate reduction group (group 2, 47 cases, 35% ≤ EF < 45%), mild reduction group (group 3, 50 cases, 45% ≤ EF < 50%) and normal group (group 4, 147 cases, EF ≥ 50%). Exclusion criteria were frequent premature beats, atrial fibrillation, and some patients could not meet the CZT SPECT gated sampling criteria (the exclusion rate was more than 10% of the total collection heart rate). All participants gave written informed consent and the study adhered to Declaration of Helsinki principles.

### Myocardial perfusion imaging acquisition protocol

Patients were underwent in CZT SPECT (Discovery NM 530c; GE Healthcare, Haifa, Israel), The patient was intravenously injected ^99m^Tc-MIBI (370–555 mBq), and rest GMPI was performed 1.0–1.5 h after injection, the total acquisition time was about 6–12 min. A array of multi-detector in the CZT SPECT system, the 19 detectors system remain in a fixed position on the cardiac volume. Per cardiac cycle was divided into 8 frames for ECG-gated data acquisition. All images were acquired (matrix size 32 × 32); energy window 20%; energy peak140 keV; pixels width 4 mm). Images were reconstructed on a standard workstation (Xeleris II; GE Healthcare) using a previously validated dedicated iterative algorithm with 50 iterations, no attenuation or scatter correction was done, but the patients with suspected diaphragmatic attenuation artifacts were collected in the prone position to improve image quality.

### Gated images analysis and phase analysis

CZT SPECT data were reconstructed using ordered subsets expectation maximization (OSEM) and analyzed by two experienced nuclear medicine physicians. The left ventricular mechanical systolic synchronization parameters were calculated using Emory Cardiac Toolbox™ (US, version 3.2) software. The quantitative parameters of left ventricular function, including end-diastolic volume (EDV), end-systolic volume (ESV) and left ventricular ejection fraction (LVEF) were obtained. Through GMPI phase analysis, 5 quantitative indicators reflection synchrony of left ventricular myocardial contraction were obtained: peak phase (PP), which is the peak of the phase diagram; phase standard deviation (PSD), which is the distribution range of the phase; phase histogram bandwidth (PHB), which is 95% width of the phase histogram; histogram skewness (HS), that is the symmetry of the histogram; histogram kurtosis (HK), which is the width from the beginning of the histogram to the peak.

### Statistic analysis

Data are shown as mean ± SD. Differences between PP, PSD, PHB, HS, HK were evaluated using one-way ANOVA. LSD-t test was used to compare differences between 2 groups. Correlation among functional and mechanical contraction synchrony factors were analyzed using Pearson test. SPSS software (IBM SPSS Statistics, version 23.0) was used for data analysis, *P* < 0.001 indicated statistical significances.

## Results

### Clinical characteristics

Clinical characteristics of the study four group patients are presented in Table 1. There was no significant difference in age (*P* = 0.431), weight (*P* = 0.626), and height (*P* = 0.336) among the four groups, but gender distribution and BMI showed significant difference (*P* < 0.001). The proportion of men in abnormal left ventricular function groups (EF < 50%) was higher than normal left ventricular function group 4 (EF ≥ 50%). The reason may be due to the fact that men have more smoking, drinking history, etc. than women. There was no significant difference in the prevalence of diabetes mellitus, hypertension, dyslipidemia, smoking, and drinking (*P* = 0.415, 0.005, 0.342, 0.020, 0.350) among the four groups.

**Table 1:** Baseline clinical characteristics among the four groups patients

Demographics	Group 1	Group 2	Group 3	Group 4	*P* value
Number of study	127	47	50	147	
Male/female	79/21	26/9	37/6	49/36	< 0.001*
Age (years)	62.2 ± 13.1	52 ± 10.4	55.8 ± 22	62.8 ± 13.6	0.431
Weight (kg)	68.3 ± 6.9	74 ± 7.1	73.8 ± 7.2	70.8 ± 14.3	0.626
Height (cm)	167.7 ± 14.6	169.7 ± 12	168.3 ± 3	164 ± 8.1	0.336
BMI (kg/m^2^)	24.3 ± 4.7	24.9 ± 3.3	25.5 ± 3	25 ± 3.7	< 0.001*
Diabetes mellitus (%)	29(22.8)	6(12.7)	7(13.6)	27(18.3)	0.415
Hypertension (%)	50(39.3)	28(59.5)	27(54.5)	88(59.8)	0.005
Dyslipidemia	127(20.8)	47(34.0)	11(15.9)	147(32.6)	0.342
Smoking (%)	49(38.5)	19(40.4)	9(18.1)	36(24.4)	0.020
Drinking (%)	25(19.6)	8(17)	2(4.5)	23(15.6)	0.350

Continuous variables are mean ± SD. Discrete variables are number (%). BMI indicates body mass index; group 1, EF < 35%. group 2, 35% ≤ EF < 45%. Group 3, 45% ≤ EF < 50%. Group 4, EF ≥ 50%

Sig., Significance

*Statistically significant finding (*P* < 0.001)

### Analysis of mechanical dyssynchrony

**Fig. 1:**
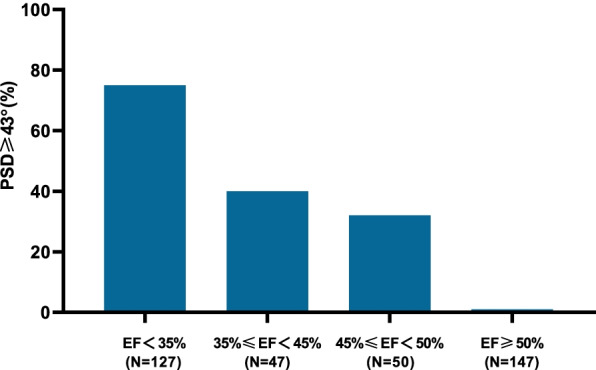
Proportion of mechanical dyssynchrony (Phase SD ≥ 43°) in each group

**Fig. 2:**
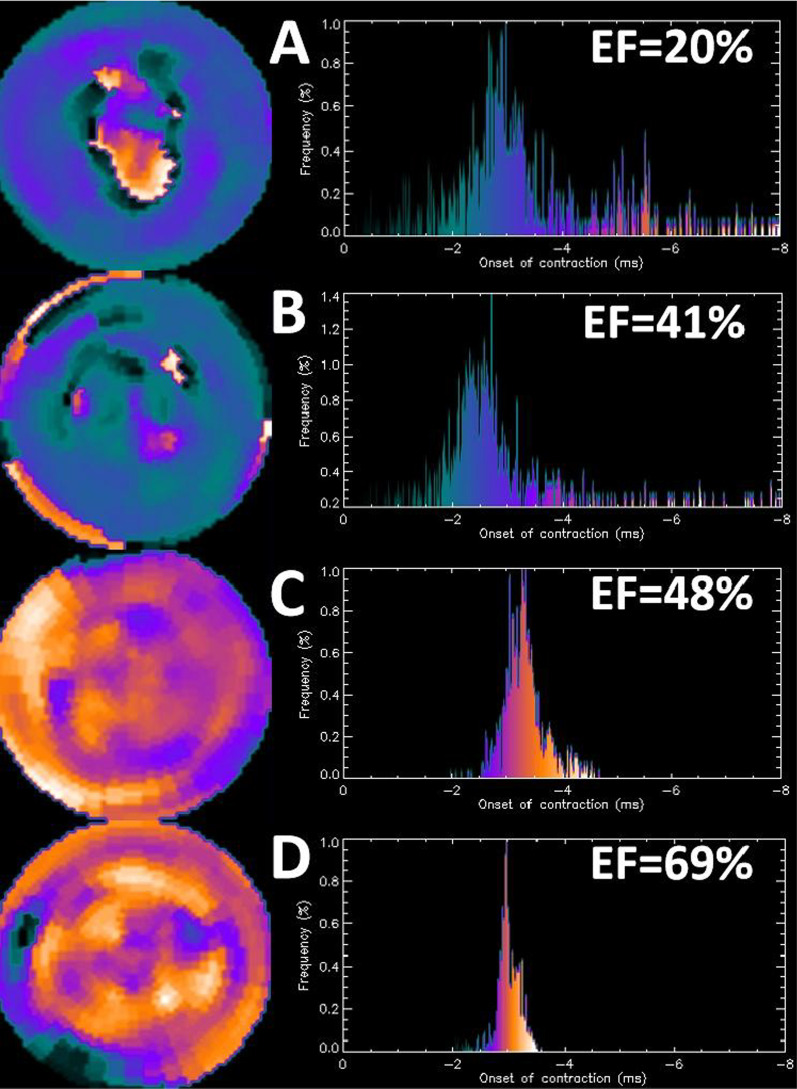
Typical examples of each group are shown. **A** Group 1, EF = 20%, EDV = 195 ml, ESV = 156 ml, PP = 133.0, PSD = 68.1°, PHB = 232.0°, HS = 2.1, HK = 4.5. **B** Group 2, EF = 41%, EDV = 120 ml, ESV = 71 ml, PP = 121.0, PSD = 52.1°, PHB = 156.0°, HS = 2.4, HK = 5.3. **C** Group 3, EF = 48%, EDV = 138 ml, ESV = 72 ml, PP = 147.0, PSD = 18.8°, PHB = 60.0°, HS = 3.3, HK = 10.7. **D** Group 4, EF = 69%, EDV = 68 ml, ESV = 21 ml, PP = 133.0, PSD = 11.3°, PHB = 34.0°, HS = 4.6, HK = 23.9

Significant mechanical dyssynchrony was defined as Phase SD ≥ 43°. 76% had dyssynchrony among patients with EF < 35% and this group has the highest proportion of mechanical dyssynchrony than the other 3 groups. Those patients with 35% ≤ EF < 45% (40% dyssynchrony), 45% ≤ EF < 50% (32% dssynchrony). The patients with EF ≥ 50% had a lowest prevalence of dssynchrony (1%) (Fig. 1). Typical cases in each group are shown in Fig. 2.

### Comparison of mechanical contraction synchrony parameters among four groups

One-way ANOVA revealed statistically significant differences in PP, PSD, PHB, HS, HK among four groups (F = 5.20, 188.72, 202.88, 171.05, 101.36, all *P* < 0.001, Table 2). PSD and PHB differed significantly between the 4 groups (*P* < 0.001); There was no statistically significant difference in HS (*P* = 0.780) and HK (*P* = 0.969) between group 2 and 3 group. There were statistically significant differences between the other groups (*P* < 0.001); There were no statistically significant differences in PP between the other groups (*P* > 0.05) except for group 1 and group 3 (t = 2.43, *P* < 0.05) or group 4 (t = 3.67, *P* < 0.001).

**Table 2:** Comparison of mechanical contraction synchrony parameters

Variables	Group 1	Group 2	Group 3	Group 4	F	*P*
PP (°)	138.2 ± 37.2	129.1 ± 20.5	123.4 ± 19.8	125.8 ± 20.3	5.20	0.002
PSD (°)	57.9 ± 18.9	41.3 ± 16.3	31.8 ± 19.4	15.3 ± 7.4	188.72	0.000
PHB (°)	184.3 ± 65.3	122.4 ± 60.1	86.1 ± 30.0	43.4 ± 15.1	202.88	0.000
HS	2.0 ± 0.5	2.8 ± 0.7	2.9 ± 0.6	4.0 ± 0.9	171.05	0.000
HK	4.6 ± 3.5	9.4 ± 5.2	9.3 ± 4.6	18.2 ± 8.8	101.36	0.000

Continuous variables are mean ± SD. Group 1, EF < 35%. Group 2, 35% ≤ EF < 45%. Group 3, 45% ≤ EF < 50%. Group 4, EF ≥ 50%

*PP* Peak phase, *PSD* Phase standard deviation, *PHB* Phase histogram bandwidth, *HS* Histogram skewness, *HK* Histogram kurtosis, *Sig.* Significance

*Statistically significant finding (*P* < 0.001)

### Correlation analysis between left ventricular function and mechanical contraction synchrony parameters

**Fig. 3:**
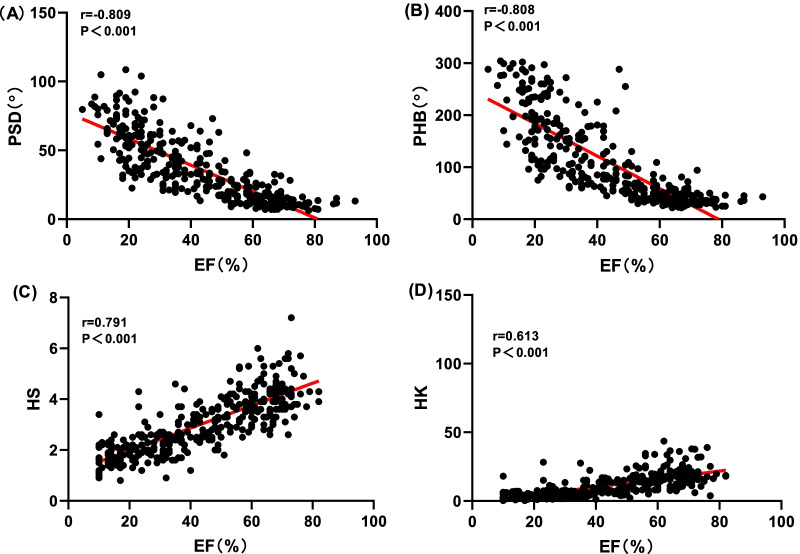
Relationships between left ventricular ejection fraction (LVEF) and mechanical contraction synchrony Parameters: **A** phase standard deviation and **B** histogram bandwidth. **C** Histogram skewness and **D** histogram kurtosis

**Fig. 4:**
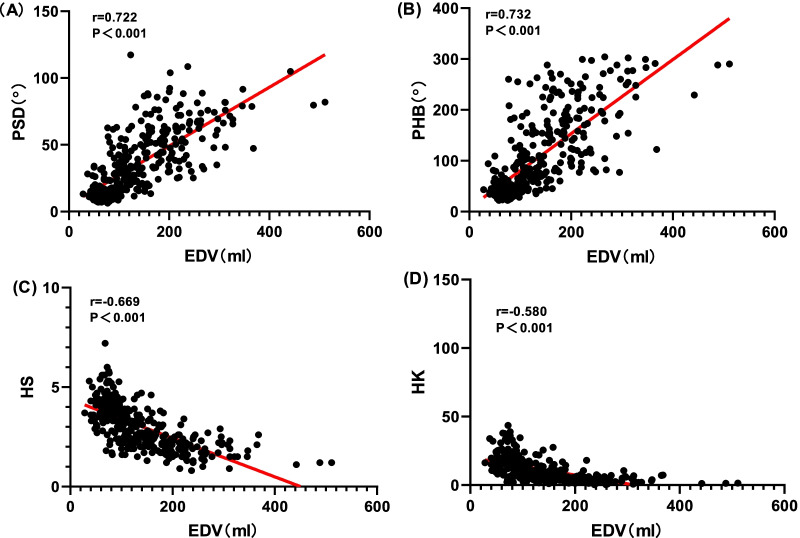
Relationships between end-diastolic volume (EDV) and mechanical contraction synchrony Parameters: **A** phase standard deviation and **B** histogram bandwidth. **C** Histogram skewness and **D** histogram kurtosis

**Fig. 5:**
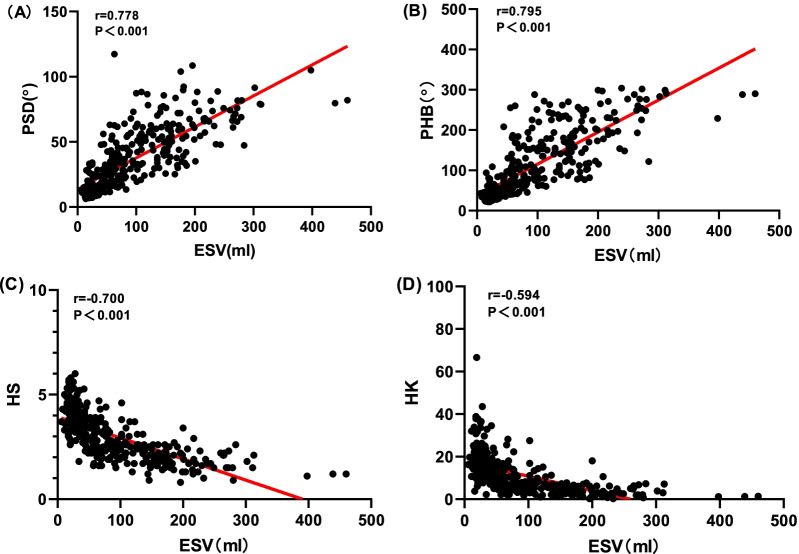
Relationships between end-systolic volume (ESV) and mechanical contraction synchrony Parameters: **A** phase standard deviation and **B** histogram bandwidth. **C** Histogram skewness and **D** histogram kurtosis

Cardiac function parameters for each group are shown. LVEF (20.76 ± 7.48)%, EDV (213.52 ± 75.12) ml, ESV (213.52 ± 75.12) ml in group 1, LVEF (39.66 ± 3.13)%, EDV (141.30 ± 41.36) ml, ESV (86.98 ± 30.95) ml in group 2, LVEF (47.68 ± 1.32)%, EDV (116.77 ± 22.14) ml, ESV (61.41 ± 12.15) ml in group 3, LVEF (63.79 ± 7.41)%, EDV (79.29 ± 21.35) ml, ESV (29.93 ± 14.22) ml in group 4. Pearson correlation analysis (Table 3) showed that PP, PSD, PHB and LVEF were negatively correlated (r = 0.194, − 0.790, − 0.799, all *P* < 0.001). Among them, PP had poor correlation with LVEF while PSD and PHB were significantly negatively correlated with LVEF, HS, HK and LVEF were positively correlated (r = 0.767, 0.676, *P* < 0.001). PSD, PHB and ESV were significantly positively correlated (r = 0.778, 0.795, *P* < 0.001), while PP and ESV were not well correlated (r = 0.145, *P* > 0.05), PSD, PHB and EDV were significantly positively correlated (r = 0.722, 0.732, *P* < 0.001), but PP had no correlation with EDV (r = 0.095, *P* = 0.078), HS, HK, EDV and ESV were all negatively correlated (r =  − 0.700 to − 0.594, *P* < 0.001) (Figs. 3, 4, 5).

**Table 3:** Correlation analysis between left ventricular function and mechanical contraction synchrony parameters

Variables	LVEF (r/*P*)	EDV (r/*P*)	ESV (r/*P*)
PP	− 0.194/0.000	0.095/0.078	0.145/0.007
PSD (°)	− 0.790/0.000	0.722/0.000	0.778/0.000
PHB (°)	− 0.799/0.000	0.732/0.000	0.795/0.000
HS	0.767/0.000	− 0.669/0.000	− 0.700/0.000
HK	0.676/0.000	− 0.580/0.000	− 0.594/0.000

*PP* Peak phase, *PSD* Phase standard deviation, *PHB* Phase histogram bandwidth, *HS* Histogram skewness, *HK* Histogram kurtosis, *EDV* End-diastolic volume, *ESV* End-systolic volume, *LVEF* Left ventricular ejection fraction, *Sig.* Significance

*Statistically significant finding (*P* < 0.001)

## Discussion

GMPI phase analysis is a new technique to quantitatively evaluate left ventricular myocardial mechanical synchronization. Phase analysis is a short axis diagram of 8 phases in one cardiac cycle from GMPI image as input information. The left ventricle is divided into more than 700 areas and 3D count distributions are extracted from each of the left ventricle short-axis data sets, then you can get phase distribution of the initial time of myocardial contraction in different parts and the whole phase of the left ventricle distribution by calculating the radioactivity count rate of each area [8, 9]. Since CZT SPECT directly converts γ-rays into electrical signals and performs meter mode acquisition, it has a higher count rate and spatial resolution. Image quality is high even in a short acquisition time with reduced injection doses [10–12]. Enough evidence has been collected from multiple laboratories in many countries and the CZT technology was considered as the ‘‘gold standard’’ of SPECT imaging [13]. CZT SPECT completes gated acquisition to obtain relevant quantitative parameters for severe heart failure, especially for patients who require CRT installation with mechanical synchrony and latest excitement evaluation. Here, 127 patients (group 1) with severely reduced LVEF had an average LVEF of 20.76 ± 7.48%, and all of them successfully completed gated collection. Studies showed that CZT SPECT (Discovery NM 530c) is consistent with conventional SPECT in detecting left ventricular mechanical contraction synchrony (98%) [14]. Cardiac function parameters measured by CZT SPECT are in good agreement with MRI [15]. The coordination and synchrony of ventricular wall motion for each segment of the left ventricular myocardium directly affects overall systolic and diastolic function of the left ventricle. Ultrasound technology application showed that the left ventricular mechanical contraction is not synchronized in the part of heart failure with preserved ejection fraction [16]. Other studies have confirmed that early heart failure is accompanied by left ventricular mechanical contraction asynchrony [17]. Based on the cardiac function classification recommended by 2018 SPECT myocardial perfusion imaging guidelines, we evaluated correlation between cardiac function and left ventricular mechanical contraction synchrony in a relatively large sample size [18]. We found that synchronization parameters of mechanical contractions in each group were significantly different among groups with different LVEF values. Pairwise comparison results indicate that phase standard deviation and phase histogram bandwidth can more sensitively distinguish among groups synchronization caused by LVEF differences, while the histogram skewness, histogram kurtosis, and peak phase suggest poorly sensitive differentiation among groups. This study found that the greater the left ventricular systolic and end-diastolic volume, the more severe the damage to the left ventricular systolic function, and the worse the left ventricular mechanical contraction synchrony. As shown in Fig. 1, larger phase distribution range brings wide bandwidth, large deflection, small skewness, and a broad asymmetrical peak phase. Jianfeng Wang et al. confirmed that LVEF negatively correlated with phase histogram bandwidth upon synchronization evaluation in patients with old myocardial infarction (r =  − 0.807) [19]. Hongbo Yang et al. found that phase standard deviation and bandwidth negatively correlated with LVEF in chronic total occlusion (CTO) lesions, while the skewness and kurtosis of the phase map positively correlated with LVEF [20], which is consistent with our findings. Note that LVEF is a powerful predictor of cardiac mortality [21]. Our study showed that the worse left ventricular mechanical contraction synchrony leads to decreased LVEF, making the systolic synchrony parameters valuable in the prediction of cardiac mortality. Hence, the role of mechanical systolic synchrony parameters should be considered for predicting the prognosis of patients belonging to the severe reduction group and moderate reduction group. PSD, PHB, HS, and HK have similar correlations with left ventricular systolic function, the worse left ventricular mechanical contraction synchrony leads to decreased LVEF, but PSD and PHB are rougher and more sensitive, suggesting that HS and HK can be used to evaluate mechanical contraction synchronization. However, larger studies are needed to determine if HS and HK are more sensitive than PSD and PHB, and if phase peak and left ventricular function parameters are poorly correlated or uncorrelated. Previous studies used PSD and PHB as the most sensitive evaluation indicators of mechanical synchrony due to their good consistency with TDI synchronization parameters. Another study showed that TDI is insufficient for detecting left ventricular asynchrony and predicting CRT efficacy [22]. Recently, synchronization evaluations based on ultrasound, including RT-3DE [23] and STI [24] have emerged and offered numerous advantages for assessing synchronization and evaluating prognosis [25]. Studies showed that conventional SPECT is consistent with RT-3DE in detecting left ventricular mechanical contraction synchrony for the patients with heart failure [26]. Future comparison studies should validate CZTSPECT sensitivity using other techniques, including RT-3DE, STI, and MRI, and evaluate the capability of HS and HK on synchronization consistency.

### Study limitations

This study has some limitations. First, we did not strictly exclude the influence of patients with occasional arrhythmia on the acquisition of synchronization parameters of ventricular mechanical contraction during the gated acquisition process. Moreover, we were unable to reach all patients for prognosis information. Future follow-ups are needed to determine the predictive effect of systolic synchrony parameters.

## Conclusion

CZT SPECT GMPI provided left ventricular mechanical contraction synchrony parameters that correlated well with left ventricular systolic function. The worse left ventricular mechanical contraction synchrony leads to decreased LVEF, making the systolic synchrony parameters valuable in the prediction of left ventricular systolic function.

## Data Availability

The datasets analyzed during the current study are available from the corresponding author upon request.
